# Production of Sustainable Construction Materials Using Agro-Wastes

**DOI:** 10.3390/ma13020262

**Published:** 2020-01-07

**Authors:** Chrysanthos Maraveas

**Affiliations:** Department of Civil Engineering, University of Patras, 26500 Patra, Greece; c.maraveas@maraveas.gr

**Keywords:** sustainable, construction materials, agricultural by-products

## Abstract

The construction sector, in modern times, is faced by a myriad of challenges primarily due to the increase in the urban population and dwindling natural resources that facilitate the production of construction materials. Furthermore, higher awareness on climate change is forcing companies to rethink their strategies in developing more sustainable construction materials. Diverse types of agro-waste ranging from rice husk ash (RHA), sugarcane bagasse ash (SCBA), and bamboo leaves ash (BLA) among others have been identified as potent solutions in the development of sustainable construction materials. In this review paper, six different construction materials, made using agro-waste products, are examined. The materials include brick/masonry elements, green concrete, insulation materials for buildings, reinforcement materials for buildings, particleboards, and bio-based plastics. The main criterion adopted in selecting the materials regards their popularity and wide-scale use in modern construction applications. Additionally, as this research emphasizes identifying alternative approaches to develop sustainable construction materials, the focus is directed toward mainstream materials whose continued use has an adverse impact on the environment. The findings obtained from the review showed that the use of agro-waste to develop sustainable construction materials was effective, as the developed materials adhered to established building standards. Therefore, this indicates that agro-waste materials have the potential to replace conventional construction materials and hence achieve economic, environmental, and social sustainability in the long run.

## 1. Introduction

A brief analysis of world population statistics highlights a steady increase in the human population, rising from 6.8 billion in 2009 to the current 7.7 billion in 2019 and an estimated 9.7 billion by 2050 [1]. On the one hand, the population increase directly indicates that health and mortality rates are improving over time, thereby leading to population growth. On the contrary, rising population levels also imply an increase in pressure levels exerted on available social amenities such as housing. As the demand for housing increases exponentially, this further strains the construction industry as well as the production of conventional materials such as cement, steel, aluminum, and wood, among others [2]. Guna et al. [3] have further argued that the production of the conventional construction materials such as cement also utilizes significant thermal and electrical energy and, as a result, translates into higher building costs.

Furthermore, such production processes account for a higher carbon footprint, polluting air, land, and water. For instance, Wi et al. [4] observe that the calcination process, employed in the manufacture of cement, requires a temperature of up to 1450 °C and also releases about 0.85 tons of CO_2_ per 1 ton of cement produced. In a separate study, Chabriac et al. [5] highlight that buildings in France account for about 23.5% of pollution from greenhouse gases (GHG) due to the utilization of conventional construction materials. In the same vein, Shafigh et al. [6] have argued that currently, the construction industry is not sustainable. These findings imply a need for more scientific research to develop construction materials that are not only more sustainable and environmentally friendly but also more affordable without compromising building quality.

On the flip side, research further reveals that the disposal of agricultural crop waste materials such as sugarcane bagasse, wheat straw, coconut, and rice husks, among others, is also a significant problem in developing nations. For instance, in India, over 600 metric tonnes (MT) of waste was reported from agricultural-based products alone [2]. Obi, Ugwuishiwu, and Nwakaire [7] further observe that with the expansion of agricultural production in the current decade, due to the intensification of farming systems, more agro-waste materials are anticipated to be produced. Madrid et al. [8] highlight that the most common strategies to manage such agro-waste include dumping in landfills, incineration, and composting, which as a result generate severe environmental concerns. Nonetheless, emerging research has shown that the re-use of agricultural waste and by-products in the development of construction materials, either in part or wholly, is a viable and tentative solution to tackle the identified challenges.

Research also shows that re-using agro-based waste helps not only to tackle the pollution problem brought about by the exploitation of conventional construction materials such as cement but also the environmental concern of disposing of the waste in landfills [9]. In an illustrative study, researchers Sathiparan and De Zoysa [10] showed that using agricultural crop wastes such as peanut shells, rice husks, rice straw, and coconut shells, as a partial replacement of sand in the manufacture of cement blocks, generated blocks that adhered to ASTM standards in terms of strength and durability features. Likewise, Ashour et al. [11] showed that the development of sustainable and non-toxic construction materials by stabilizing soils using natural wheat and barley straw fibers generated reinforced bricks with impressive thermal and static qualities. Other researchers such as Chaussinand, Scartezzini, and Nik [12] further illustrated that developing buildings using only straw bales and soil led to the generation of more sustainable buildings. The researchers argued that this arose due to the optimal features of the materials, such as low embodied energy and high thermal performance. In this regard, the findings confirm that using agro-waste in developing construction materials, either in whole or partly, helps tackle the sustainability challenge while reducing pollution and adverse environmental effects.

In this review paper, a detailed examination of the use of agro-based waste in the production of construction materials, both in part and wholly, is presented. A discussion of their advantages and disadvantages over conventional construction materials is further undertaken in order to identify possible research gaps for further development. The paper is organized into five sections. The first examines the impact of the construction industry on environmental sustainability. The second section outlines the novelty of the research where the unique contribution of this research is identified. Several seminal review papers on the research area are examined critically, in this section, in an effort to identify why this research is necessary. The third expounds on the use of a different type of agro-waste in the manufacture of diverse, sustainable construction materials, such as bricks, green concrete, reinforcement materials, and bio-based plastics, among others. The fourth section further explores the advantages and disadvantages of using the materials. The fifth section undertakes a discussion on the different findings identified in the paper, while the sixth section presents a conclusion.

## 2. Novelty of the Research

Over the years, substantial work has been undertaken on the use of agro-waste in developing sustainable construction materials. A confluence observed from different research studies is that they all focus on tackling two generic problems; the first regards reducing the impact of the construction industry on climate change through promoting the use of alternatives. One example is using agro-waste such as sugarcane bagasse ash (SBA) in the place of cementitious materials, thereby eliminating the release of GHGs from production processes. The second pertains to resolving the problem of disposing agro-waste sustainably, whereby there is an emphasis on avoiding waste disposal through landfills due to their adverse environmental and health impacts. This research is no different, as it also focuses on the two generic problems.

However, on the contrary, an in-depth examination of seminal research papers reveals various significant gaps that are addressed in the current paper. The first pertains to the narrow scope associated with the review papers compared to the comprehensive examination undertaken in this research. The review of Rahman et al. [13,14,15,16] shows that the researchers focus on the sustainable development of bricks [16], masonry blocks [15], and self-compacting concrete [13]. In contrast, this review examines a broad range of construction materials, including brick/masonry elements, green concrete, insulation materials for buildings, reinforcement materials for buildings, particleboards, and bio-based plastics. Therefore, this research provides greater insight on the use of agro-waste in broader applications in the construction industry as opposed to focusing on a limited number of areas. The reader is subsequently able to identify, at a glance, how different types of agro-waste can be used in developing a wide range of construction materials. To the best of the researcher’s knowledge, this is the only paper that has tried to provide a comprehensive review of agro-waste in the development of a broad range of construction materials, ranging from bricks to concrete and reinforcement materials.

Secondly, Rahman et al. [16] observed that there lacked adequate guidelines to facilitate the incorporation of waste materials during the development of bricks. Nevertheless, the findings obtained showed that fly ash bricks improved significantly after adding 10% palm oil fuel ash (POFA), while the highest compressive strength value was also obtained when fly ash and POFA were added at a ratio of 1:1. The lack of guidelines in incorporating waste materials is also emphasized in the current research; however, unlike previous studies [16], the paper provides broader insight on how the ratios of agro-waste to conventional materials influence their different features. For instance, this paper answers the question: how does the percentage of agro-waste vary in the production of bricks/masonry components vis a vis reinforcement and insulation materials as well as green concrete?

Thirdly, in each of the different review studies, researchers have been observed to emphasize the advantages of incorporating agro-waste in developing construction materials. Ting et al. [17] reported that the integration of POFA in the production of sustainable concrete led to advantages such as reduced production costs and an improved performance of concrete both in its fresh and hardened states. POFA was reported to enhance the workability or homogeneity of fresh concrete as well as the compressive strength, tensile strength, permeability of water, and modulus of elasticity [17]. Rahman et al. [15] reported similar findings, revealing that the incorporation of POFA in the development of masonry components improved their compressive strength and load-bearing capacities, leading to adherence to the Malaysian Standard MS76:1972. However, despite the emphasis on the benefits of using agro-waste, the researchers have been observed to fail to identify the shortcomings of using these materials. Subsequently, this research aims to bridge this gap by critically examining the disadvantages of agro-waste in developing sustainable construction materials. The paper seeks to answer the question: Do the advantages of incorporating agro-waste in building materials outweigh their disadvantages? Do solutions to the identified shortcomings exist? Furthermore, the research also seeks to establish whether agro-waste-based construction materials adhere to building codes and standards in different contexts, such as for instance using sugarcane bagasse ash (SBA), rice husk ash (RHA), or POFA. As such, it helps answer the question: Does the use of different agro-waste materials influence their adherence to building standards?

Fourth, the various review studies, Rahman et al. [13,14,15,16] and Ting et al. [17], have also been observed to focus only on the integration of only one type of agro-waste material in developing sustainable construction materials. Ting et al. [17] investigated the use of POFA in enhancing the qualities of concrete, while Rahman et al. [13] used rice husk ash (RHA) in the development of self-compacting concrete. Rahman et al. [14] further used peat oil clinker (POC) in improving the engineering properties of peat, and in a separate research incorporated POFA in developing masonry components [15]. An observation from the different studies is that the researchers focus on incorporating only one type of agro-waste in developing the construction materials. A difference is only observed in a study by Rahman et al. [16] where fly ash and POFA are incorporated in developing blended bricks [16]. The present study seeks to advance this research area by shedding further light on studies that have adopted a similar approach. As such, the study will answer the question: Does the incorporation of more than one type of agro-waste material influence the overall qualities of the construction material? Are there any advantages or disadvantages that arise from adopting such an approach?

Therefore, this research is unique, as it not only provides a comprehensive review of the use of agro-waste in the production of a wide range of construction materials, but also provides insight on the influence of using different ratios in combining agro-waste as well as the incorporation of more than one agro-waste material in the development of sustainable construction materials. Additionally, it also discusses the shortcomings of using agro-waste and pinpoints areas where future work can be undertaken. The paper also details important research gaps that can be developed further in future applications.

## 3. The Construction Industry and Environmental Sustainability

A prerequisite to examining the different applications of agro-waste in the development of construction materials is briefly reviewing the construction industry and its impact on environmental sustainability. According to Statista, the total expenditure in the construction industry (residential and non-residential), in both the private and public sector, was valued at $11.4 trillion in 2018 [18]. The value represents a steady increase in expenditure from $10.9 trillion in 2017 and $10.5 trillion in 2016. Furthermore, the report estimates an increase in expenditure to $14 trillion by 2025 [18]. Directly, this implies that more resources are being directed toward the development of the built environment, either infrastructure such as roads or residential and commercial buildings. In effect, this translates to the utilization of more conventional raw materials such as cement and sand. The World Bank reiterates similar sentiments, adding that the construction industry is the largest global consumer of raw materials with the constructed environment accounting for 25% to 40% of the world’s total emission levels of greenhouse gases [19]. The report also highlights that the industry is anticipated to grow by 4.2% on an annual basis between 2018 and 2023. The growth is regarding market value through the expansion of residential, non-residential, and infrastructural construction projects [19]. PwC also echoes similar sentiments in its Global Construction 2030 report, which posits that the construction output is estimated to grow by 85% to reach $15.5 trillion worldwide by 2030 with India, the US, and China accounting for about 57% in global growth [20].

From the analysis of these growth statistics, two key findings are apparent. First, as expenditure in the industry increases in the future, more raw materials (sand, cement, etc.) are expected to be consumed [18]. Second, based on the findings from the World Bank report, it was revealed that high emissions of greenhouse gases are attributed to construction activities and developed buildings. As such, it is also expected that in the future, such high emissions will also continue to be witnessed [19]. Additionally, the UNEP 2018 Global Status report reveals that in 2017, construction operations and building construction accounted for 36% of the global use of energy and up to 40% of carbon dioxide emissions [21]. Similarly, research by Willmott Dixon on the impact of construction and the built environment reported that the construction industry was ranked as one of the least sustainable sectors due to its high dependence on non-renewable resources such as water (50%), energy (45%–50%), the loss of agricultural land (80%), and building materials (60%) among others [22]. The report also revealed various global pollution estimates attributed to buildings such as air pollution (23%), climate change (50%), landfill waste (50%), the pollution of drinking water (40%) and the depletion of the ozone layer (50%) [22].

The corollary of the two aspects (the depletion of non-renewable resources, high pollution) implies a renewed emphasis on the need for sustainability in the construction industry. On the one hand, there is a need to ensure raw materials extracted for construction purposes such as cement and sand are not depleted, while on the other hand, it is also vital to ensure the deliverables from the construction industry (buildings and infrastructure) do not generate high carbon emission levels. Subsequently, over the years, various initiatives have been launched, and numerous research studies have been conducted in order to ensure that the construction industry is sustainable. The UNEP 2018 Global Status report reveals that international agreements are providing the much-needed direction toward achieving sustainability in the industry [21]. For instance, international dialogue on climate change is undertaken by countries signed under the United Nations Framework Convention on Climate Change (UNFCC), where submissions on nationally determined contributions (NDCs) relating to improvements on buildings are made [21]. As an example, Canada submitted NDCs with new targets for the building sector, including aspects such as building codes that were net-zero and energy ready. Additionally, other global alliances such as the Global Alliance for Buildings and Construction (GlobalABC) also support countries to enhance the reduction of emissions in the building and the construction sector [21].

On the other hand, scientific research is also revealing novel approaches that promote sustainability in the construction industry. One of the characteristics of sustainable or green buildings is their use of technology-based strategies to promote the conservation of water, energy, and materials [23]. For instance, these strategies include using energy-efficient equipment, alternative energy sources, and low-embedded energy materials. A second approach regards the development of building materials from agro-waste, as revealed by diverse researchers [10,11,12]. Relying on agro-waste to develop building materials facilitates the reduction of pollution that is rife in the construction industry and also helps tackle the challenge of waste disposal, which troubles the agricultural processing industry.

## 4. Development of Construction Materials from Agro Waste

In this section, a discussion on the development of different construction materials from agro-waste materials is undertaken.

### 4.1. Bricks/Masonry Components

Bricks as masonry components have been predominantly used in the construction industry since the early centuries [9]. Traditionally, the brick-making processes involve three fundamental steps: mixing the raw materials (earth-based materials such as clay and water), brick molding and drying, and finally, firing them to obtain appropriate strength. A significant shortcoming of this brick production process results in the generation of substantial amounts of greenhouse gases. Luby et al. [24] reveal that high pollution arises from brick kilns, which rely on coal technology, being primarily used to fire the bricks. Interestingly, their research on why such technology is still relied upon in Bangladesh reported that in most instances, brick buyers preferred bricks made from such kilns as opposed to more modern ones due to their lower costs. Similarly, kiln owners used them since they were able to generate a significant return of investment [24].

**Figure 1:**
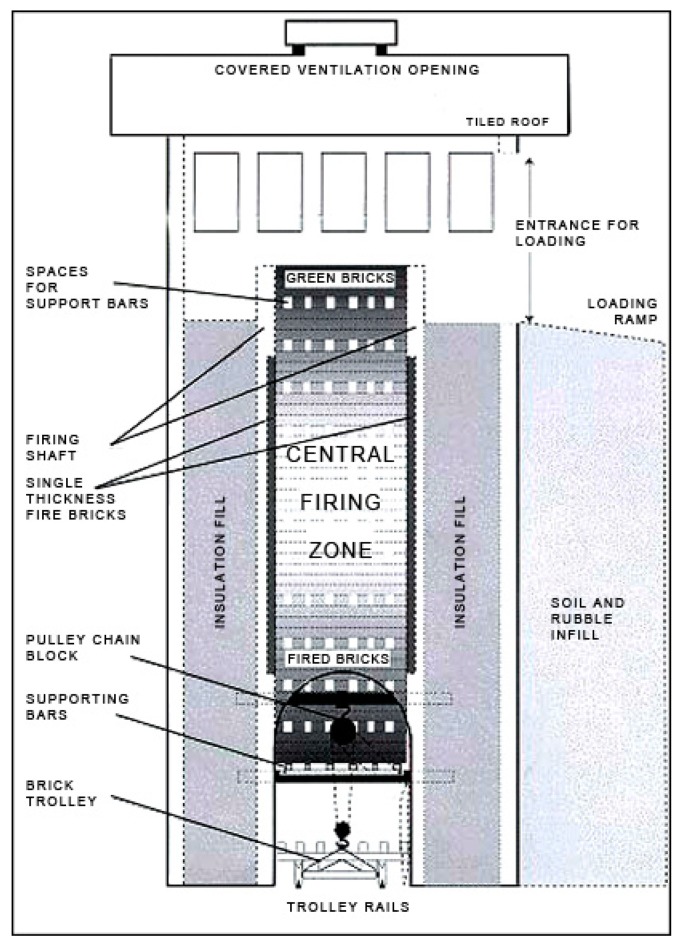
Modern vertical shaft brick kiln (VSBK) kiln schematic diagram [26].

A second disadvantage of the process stems from their excessive usage of non-renewable materials such as water and clay, thereby facilitating the depletion of natural resources [9]. It is important to invest in modern kilns to alleviate such issues in order to enhance the efficiency of the carbonization process, thereby reducing emission levels. Gomes and Hossain [25] highlight the Hoffman Kiln (HK) and the vertical shaft brick kiln (VSBK) as two readily available alternatives that have been observed to reduce emission levels by 42% and 29% respectively as compared to the traditional bull’s trench kiln (BTK). Figure 1 below illustrates the schematic diagram of a VSBK kiln.

**Figure 2:**
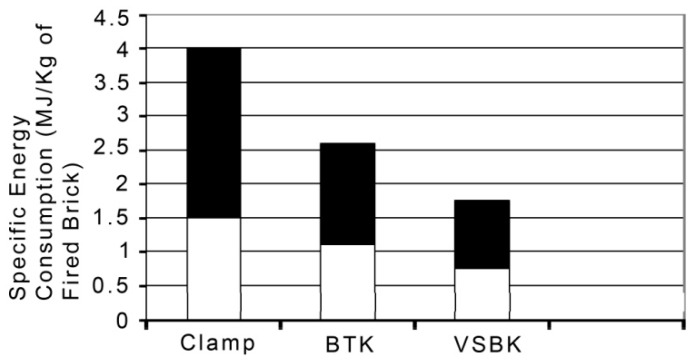
Energy consumption of VSBK, bull’s trench kiln (BTK) and clamp kilns [26].

According to Daraina et al. [26], the vertical shaft brick kiln (VSBK) technology originated from China. A comparative evaluation of the VSBK against clamps and the traditional BTK kilns reveal various advantages over clams and BTKs such as lower construction costs, compactness of the kiln, high energy efficiency in firing bricks, and weatherproof nature, as it can be operated even during the monsoon periods. The energy consumption in the VSBK is also reasonably lower than the clamp and BTK technologies, as revealed in Figure 2 below. The VSBK consumes about 0.57 Gcal/’000 nos brick, while the free chimney and fixed chimney BTK consume 1.08 and 0.86 Gcal/’000 nos brick, respectively.

However, a more effective strategy regards the transition to more sustainable brick-making processes. The processes incorporate agricultural waste products, adding useful properties in the bricks and also helping tackle the waste disposal challenge associated with agricultural processing [27]. Kazmi et al. [27] utilized rice husk ash (RHA) and sugarcane bagasse ash (SBA) in producing clay bricks, by incorporating 5% of the RHA and SBA by clay weight. The findings obtained showed that the bricks’ compressive strength and modulus of rapture decreased as higher quantities of RHA and SBA were incorporated into the clay burnt bricks. However, on the contrary, the modulus of rupture and compressive strength of the bricks adhered to Pakistan building codes and ATSM standard guidelines. Likewise, the developed bricks were observed to be light weight in nature, thereby reducing the structural load of the buildings. Further analysis also showed that they were resistive to weather, and efflorescence resistance was also enhanced by incorporating the RHA and SBA. Examination under a scanning electron microscope (SEM) further showed that the bricks were also more porous, thereby improving the overall environmental sustainability of buildings.

In a separate study, Kizinievič et al. [28] utilized oat husk and barley husk and middlings as an additive in firing clay bricks, and after that, assessed the mechanical, porosity, and physical qualities of the developed bricks. Brick molding components were prepared by adding three separate concentrations of the oat or barley husks and middlings: 5%, 10%, and 20%. The temperatures were also set to 900 and 1000 °C and maintained for an hour. The findings obtained showed that the highest quality eco-friendly clay bricks were obtained where the solid wastes were added at a concentration between 5% and 10% [28]. The bricks had a density of 1300 to 1800 kg/m^3^, total open porosity between 34% and 49%, water absorption rates between 14% and 28%, and compressive strength of 3.3 to 9.5 MPa. An observation made was that the solid wastes (oat, barley husks, and middlings) incinerated at a temperature of 500 °C, thereby forming a porous structure in the clay body. A comparison of the techniques adopted by Kazmi et al. [27] and Kizinievič et al. [28] in using agricultural waste as additives in making clay bricks showed that only a minimal amount of the waste was used (5%–10%). Likewise, similar brick features were also identified—there was a higher porosity in both cases, excellent compressive strength, and high density despite the utilization of various agricultural solid waste (rice husks and sugarcane bagasse [27] and oat/barley husks and middlings [28]).

In a third study, Taurino et al. [29] focused on the manufacture of light-weight bricks by integrating wine wastes (WWs) such as wine less, grape seeds, and stalks with clay. Similar to the previous studies, the researchers varied the concentration of WWs and evaluated the bricks that were generated. The findings obtained showed that bricks with the highest mechanical and physical properties were produced from the use of 5% weight of the wine or less. Similarly, the density of the bricks was also reduced by up to 13% based on the concentration of WWs used. The flexural strength of the bricks also decreased based on the concentration of WWs used.

Interestingly, the researchers [29] argued that the increase in porosity of the bricks led to a commensurate improvement in their light weightiness and thermal insulation properties, thereby becoming suitable for application in green buildings. A side-by-side comparison of the findings against previous studies [27,28] highlights that a similarity in the concentration of waste materials influenced the overall qualities of the bricks, and additionally, the highest brick qualities were attained at a low concentration (5%). Nonetheless, a difference that exists is the use of wine waste products, which indicates a shift from the more traditional agricultural food products such as oats and rice.

De Silva and Perera [30] further investigated the impact of adding waste rice husk ash (RHA) on the thermal and acoustic performance of fired clay bricks. In the study, the clay was mixed with six different concentrations of RHA wastes (0%, 2%, 4%, 6%, 8%, and 10%). Bricks measuring 195 mm × 95 mm × 50 mm were further prepared and fired in an industrial kiln, and various properties were examined, including physical properties, such as the distribution of particle sizes and Atterberg’s limits, material chemical composition, thermal and acoustic performance, and compressive strength. The findings obtained showed that optimal brick qualities were obtained at 4% RHA and included better compressive strength at 3.55 N/mm^2^, which was 32% more improved compared to traditional clay fired bricks, and had a high water absorption rate at 19%. Furthermore, the bricks demonstrated better indoor temperature reduction by 6 °C and noise reduction by 10 dB compared to conventional clay bricks. Comparison of the technique utilized by De Silva and Perera [30] to that of Kazmi et al. [27], who also used RHA as a waste additive in developing clay bricks, revealed that only a minimal concentration of the solid wastes is essential in enhancing the brick properties—5% and 4% RHA, respectively. Likewise, the findings in both instances reveal that the structural properties (compressive strength) and porosity of the bricks were enhanced by using RHA waste.

Deraman et al. [31] also investigated the impact of utilizing empty fruit bunch (EFB) and coconut fiber (CB) as an additive poring agent in the production of clay bricks. The research utilized three concentrations of the waste products—0%, 5%, and 10%—and after that, an evaluation of the physical–mechanical properties of the bricks, such as the compressive strength, thermal conductivity, and water absorption rates was undertaken. The findings showed that where 5% of EFB was added, the bricks adhered to the minimum requirements of the BS392:1985 in terms of water absorption and compressive strength as well as the ASTM C517 thermal conductivity standard. Subsequently, the study concluded that the bricks were a potential solution in enhancing the thermal qualities of building envelopes.

Other researchers have considered the use of other agro-wastes (not necessarily food wastes) such as sawdust as an additive agro-waste product from timber-related activities in brick production. For instance, Mandal, Verma, and Sinha [32] blended different concentrations of fly ash (FA) and red mud (RM) with sawdust in the preparation of insulation bricks. An examination of the effects of the FA to RM ratios, firing temperatures, and sawdust blending on the physical characteristics of the bricks were also conducted. The findings showed that bricks with the highest maximum strength had an FA to RM ratio of 60:40 independent of the firing temperature. Furthermore, the results also showed that increasing the firing temperature enhanced the strength of the bricks, whereas the use of sawdust blending enhanced the porosity and thermal insulation of the bricks [32]. Bricks that were made by blending sawdust (7.5%) and 40% red mud at 1100 °C were able to meet the criterion of Type A insulation bricks specified by IS:2042.

In a separate study, Muñoz et al. [33] developed fire clay bricks by incorporating grapevine shoots as a pore-forming agent. In the study, the researchers used wood chips from pruned grapevine shoots as an additive element in the manufacture of fired clay bricks (FCB). The focus of the study was to assess the effect of the agro-wastes on the particle sizes of the bricks where three sizes were considered: 0–0.5 mm, above 1.5 mm, and 0.5–1.5 mm. The mechanical and thermal characteristics of the bricks were also evaluated. The findings showed that a maximum of 10% wood chip content generated bricks with the highest compressive strength and water absorption rates, while the thermal conductivity was reduced by up to 50%. A comparison of Muñoz et al. [33] against Mandal, Verma, and Sinha [32], who also used non-food related agro-wastes in making bricks, reveals a similarity in that only a minimal percentage of the waste products led to the high-quality bricks with optimal compressive strength and water absorption rates. Thermal features were also enhanced in both instances.

### 4.2. Common Approaches toward Green Concrete

In addition to bricks, concrete is also highly used in the construction industry. Prusty et al. [34] observe that concrete comprises a mixture of cement, fine aggregate, and coarse aggregate, all of which are derived from natural resources. Subsequently, as the demand for housing increases, significant pressure is mounted on the non-renewable natural resources, thereby sparking research on the use of alternative agro-waste materials to produce concrete.

To begin with, Modani and Vyawahare [35] investigated the effect of replacing untreated sugarcane bagasse ash (SCBA) by a volume of fine aggregate in concrete in ratios of 0%, 10%, 20%, 30%, and 40%. The water–cement ratio was also maintained at 0.40, and the dose of the superplasticizer was maintained at 0.8%. After that, the casted concrete specimens were cured under standard laboratory conditions and tested for 7 days and 28 days for compressive strength, sorptivity test, and tensile strength. The findings obtained showed that with the compressive strength, a specimen that contained a 10% replacement of the SCBA demonstrated better results than those that had 0% SCBA. Additionally, a further increase in SCBA led to a decrease in compressive strength and a decline in the properties of fresh concrete. An increase in the strength of the mixes with SCBA was recorded at longer time periods due to pozzolanic properties.

**Figure 3:**
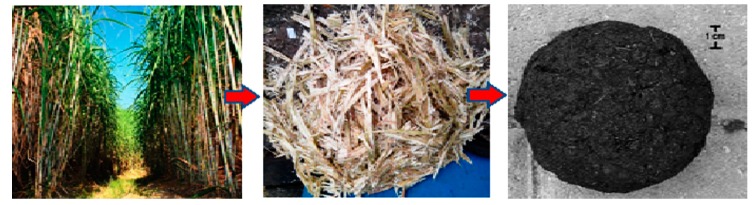
Processes to produce sugarcane bagasse ash [34].

Further tests also showed that the tensile strength and sorptivity decreased as the level of SCBA was increased. The results of the study have a significant economic implication, since they indicated that SCBA could be used as a viable alternative to aggregates in concrete production. Prusty et al. [29] have also weighed on the issue, revealing that in India, about 10 million tons of sugarcane are treated as waste, and as such, suitable conversion and application as construction materials facilitate their disposal. Figure 3 below illustrates the conversion process from raw sugarcane to bagasse and finally sugarcane bagasse ash.

**Figure 4:**
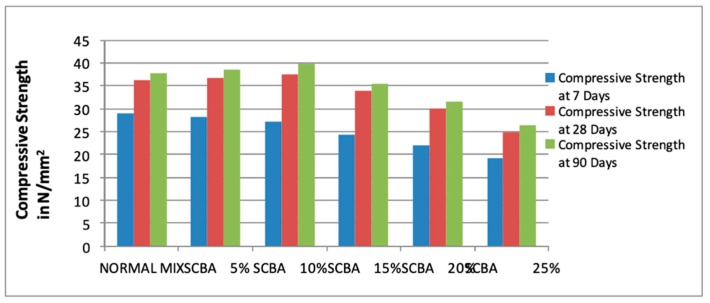
Compressive strength for the concrete mixes after 7, 28, and 90 days [36].

Rao and Prabath [36] also investigated the effect of partially replacing bagasse ash by the weight of cement in concrete production in ratios of 0%, 5%, 10%, 15%, and 25%. First, the researchers ground the bagasse ash until particles could pass the 90 µm sieve, reaching the specific surface area of 4716 cm^2^/gm. After that, Portland cement was replaced by the ash at the specified ratios, and the water–cement ratio was maintained at 0.42 while the cement content remained at 378 kg/m^3^ for the control mix. Tests for the compressive strength, split tensile strength, and flexural strength was also undertaken for cured concrete at 7, 21, and 90 days, respectively. Findings obtained showed that the cement mix with 10% bagasse ash demonstrated the best results, such as higher compressive and flexural strength after 90 days [36]. Figure 4 below displays the results of the compressive strength for the different mixes at 7, 28, and 90 days.

Additionally, from the figure, it was revealed that the compressive strength of the SCBA mixes at 7 days decreased gradually as the ratio was increased. At 28 days, the compressive strength gradually increased up to 10% SCBA, and after that, it decreased gradually with the increase in the SCBA ratio. A side-by-side comparison of Rao and Prabath [36] against Modani and Vyawahare [35], who also utilized SCBA, reveals both differences and similarities. A noteworthy similarity in the two studies stems from the fact that the best quality bricks were obtained at a 10% SCBA ratio, thereby indicating that only minimal agro-waste percentage was required in producing high standard concrete. However, several differences emerged; first, it was observed that Modani and Vyawahare [35] replaced SCBA by volume of fine aggregate in concrete, whereas Rao and Prabath [36] replaced SCBA by volume of cement. Second, it was also observed that with Rao and Prabath [36], curing was undertaken for three different periods—7, 28, and 90 days—while Modani and Vyawahare [35] only cured the concrete for 7 and 28 days. The two differences have a significant effect on the economic implication of using agro-waste in concrete production, as they indicate that SCBA can be both used as a replacement of fine aggregates and cement. Furthermore, the findings by Rao and Prabath [36] that curing for 90 days produced better results than 28 days is also essential for practitioners in the construction industry who benefit from such knowledge.

In a separate study, Rodier et al. [37] investigated the impact of adding sugarcane bagasse ash (SCBA) and bamboo leaves ash (BLA) on the pozzolanic and hydration qualities of cement pastes. Electrical conductivity measurements were utilized in investigating the pozzolanic activity of the binary and ternary ash mixtures, whereas the pozzolanic reaction between calcium hydroxide solution and the ashes was quantified using a kinetic–diffusive model. Additionally, mechanical tests, X-ray diffraction, isothermal calorimetry, and thermal gravimetry analysis were also used in examining the impact of ashes on the hydration of the different cementitious pastes. The findings obtained showed that ternary mixtures of BLA and SCBA had higher pozzolanic activity than the binary mixture of SCBA only [37]. Furthermore, it was also observed that higher compressive strengths were recorded for the binary and ternary mortars than the control mortar, which did not have such additives. Similar to Rao and Prabath [36], who replaced SCBA by volume of cement, the results in the study showed that the replacement of cement by 10% weight of SCBA and BLA provided optimal features of the cement. However, a notable difference observed was that the incorporation of BLA to the SCBA improved the compressive strength of the mortar. Directly, this suggests that manufacturing green concrete by replacing cement by more than one type of agro-waste material has a positive impact on the features of the concrete. Rodier et al. [37] also revealed that less energy was consumed during the production of 1 tonne of binder using agro-industrial ashes as compared to conventional cement.

Akram et al. [38] further investigated the use of sugarcane bagasse ash (SCBA) as a viscosity-modifying agent in self-compacting concrete. According to the researchers, self-compacting concrete has received significant attention from different practitioners, since it eliminates the need for a vibrator during compaction. However, its adoption and usage are hindered by its relatively higher cost of supply over conventional concrete. To that end, the study sought to investigate the relative costs and the performance of using sugarcane bagasse ash (SCBA) as a viscosity-modifying agent in self-compacting concrete. Important variables considered included the water–binder ratio, proportion of bagasse ash, and dosage of superplasticizer to handle flowability. The cement and water content was held constant. The findings obtained showed that compressive strengths developed by self-compacting concrete mixes with bagasse ash at 28 days were comparable to the control concrete. Additionally, the study showed that the overall costs of ingredients of the specific concrete mixes were 35.63% lower than the control concrete, while both had a higher compressive strength of 34 MPa [38]. The significance of the study is that it highlights that agro waste can be adopted as an ingredient in the production of self-compacting concrete in addition to ternary and binary mixes used in conventional concrete applications.

Other researchers have also investigated the use of other agro-wastes, such as groundnut shells, in the production of green concrete. For instance, Kimeng et al. [39] investigated the use of groundnut shells as a partial or full replacement of fine aggregate in the production of light concrete panels in Nigeria. A total of 63 samples of concrete were cast using the groundnut shell replacements in seven different ratios: 0%, 10%, 20%, 30%, 50%, 70%, and 100%. Compressive strength tests, as well as density measurements, were taken after 7, 14, and 28 days. The findings obtained showed that where groundnut shells were only used as aggregates, the density of the blocks was recorded as 830kg/m^3^, whereas where sand aggregates were used, the density increased to 2160 kg/m^3^. Further results showed that the average compressive strength for 0% to 100% ground shells was recorded at 5.83 N/mm^2^ to 0.9 N/mm^2^ at 7 days, 8.07 N/mm^2^ to 0.5 N/mm^2^ at 14 days, and 10 N/mm^2^ to 0.6 N/mm^2^ at 28 days. The moisture absorption rates also increased from 0.47% to 2.04%, respectively. The findings also showed that 30% to 70% replacement of the aggregates generated positive findings. However, it was observed that the groundnut shell panels could not be utilized in structural applications due to their lower compressive strength, but they were efficient in non-load bearing wall partitions [39]. The findings from the study highlight a potential economic value of partially using groundnut shells as a replacement of aggregates in concrete production, especially since the disposal of groundnut shell waste is highly problematic in Nigeria. The potential of using waste products is that farmers are able to benefit from the sale of both groundnuts and groundnut shells, which are the waste products.

**Figure 5:**
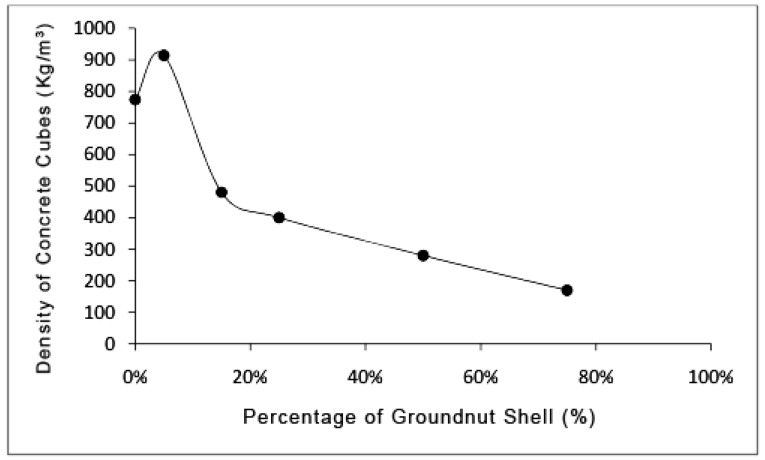
Compressive strength variation based on groundnut shells percentage levels [40].

Sada et al. [40] also investigated the use of groundnut shells as a replacement of fine aggregates (river sand) in concrete manufacture in Nigeria. In the study, concrete slabs of dimension 150 × 150 × 150 mm were cast at replacement levels of 0%, 5%, 15%, 25%, 50%, and 75%. Tests for the compressive strength, density, and slump of the concrete mixes were after that assessed after 28 days. The findings showed that at the 5% replacement level, the highest compressive strength of 40.59 N/mm^2^ and density of 2533.33 kg/m^3^ were recorded. However, a further increase in the proportion of groundnut shells beyond 5% led to a decrease in both the compressive strength and density of the concrete slabs. For instance, at 75% replacement, the concrete slabs registered a compressive strength of 7.56 N/mm^2^ and a density of 1854.81 kg/m^3^. A similar conclusion to Kimeng et al. [39], who also used groundnut shells as a replacement of aggregate in green concrete production, was identified whereby Sada et al. [40] revealed that groundnut shells had potential in the manufacture of light-weight concrete. Figure 5 below displays the variation in compressive strength based on the levels of groundnut shells utilized.

### 4.3. Insulation Materials for Buildings

**Figure 6:**
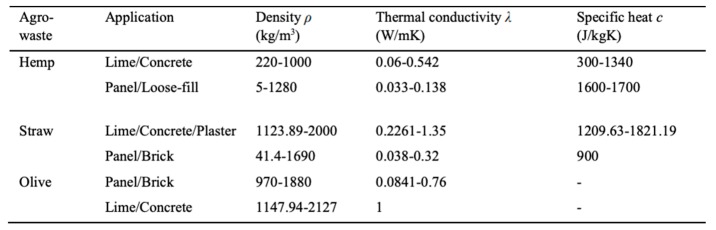
Thermal properties of biomass residues [41].

Agro-wastes have also been deployed in the manufacture of insulation materials for buildings. According to Liu et al. [41], the use of biomass such as agro-residues, economical plants, and forest residues in thermal insulation of buildings has received increased interest in the last few decades. The researcher also revealed that a wide range of agro-waste materials has also been deployed in thermal insulation—materials such as hemp, straw, coconut, wood, and flax were highly popular, while others such as sisal, reed, grass, and pineapple were rarely used [41]. Figure 6 below presents a summary of the thermal properties of prevalent biomass residues.

As observed in Figure 6, the thermal properties of the residues, such as their conductivity, heat capacity, and thermal diffusivity generated the highest research interest, followed by their density. Other factors such as compressive strength and water absorption were also considered, while properties such as pH and radioactivity were not highly emphasized. In the same vein, the research also showed that different manufacturing approaches were also utilized where bonding by using a binder to make different materials was highly used [41]. In other instances, the use of agro-wastes in their natural form, for instance, the packaging of straw bales and high pressing to develop other loose materials, were adopted.

**Figure 7:**
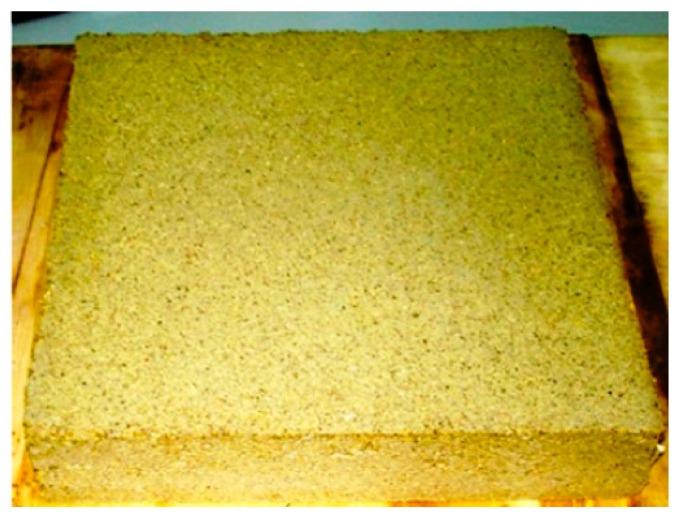
Lime–hemp mixture in insulation panels [42].

In separate research, Benfratello [42] investigated the thermal qualities of insulation panels built using hemp only and a mixture of hemp and concrete, with the emphasis being directed to the amount of fiber added and the granulometry of the mixture. Findings from the analysis showed that the biocomposite had excellent insulation properties and mechanical resistance. However, it was lighter than traditional concrete and could only be used in an appropriate way where lower loads in buildings where required, for instance, in the green coverings of the top of an existing building. Figure 7 below illustrates the lime–hemp mixture created.

An earlier study by Elfordy et al. [43] had also investigated the thermal and mechanical properties of lime–hemp concrete blocks. Mechanical aspects such as the flexural strength, hardness, and compression strength were evaluated, and density within the blocks also measured. The findings showed that the increase in mortar density resulted in a commensurate increase in the thermal conductivity and mechanical features of the blocks. An interesting finding from the side-by-side comparison of Benfratello [42] and Elfordy et al. [43] confirms that hemp, as a biomass residue, provides excellent thermal insulation properties for buildings. However, an additional finding by Elfordy et al. [43] means a need to strike a balance between achieving good thermal insulation and leveraging on the mechanical features of the developed composites, since an increase in density led to the enhancement of compressive strength, hardness, and flexural strength. The findings also reiterate Liu et al. [41], who observed that hemp was highly popular as a thermal insulator in building materials.

Aside from hemp, straw has also been widely adopted in the manufacture of thermal insulation materials for buildings. A study by Rojas et al. [44] investigated the use of natural fibers from corn husk and wheat straw as an alternative to insulation materials fabricated from petrochemicals. In order to investigate the thermal conductivity and density of the composites, the Taguchi method was employed, with four control factors being evaluated: boiling time, fiber length, blending time, and concentration of NaOH in an L-9 orthogonal array. The Taguchi method deploys a particular set of orthogonal arrays, which recommends a minimum number of experiments that generate maximum information for different factors that affect the outcomes [45]. Additionally, the flexural and compressive strengths of the composites were also measured and compared against polystyrene insulation blocks. The findings obtained showed that the blocks demonstrated good thermal conductivity between 0.046 and 0.047 W/mK, while the mechanical features such as flexural stress were comparable to the expanded polystyrene blocks [44]. The positive results have a significant economic implication in developing countries, such as Chile, since natural fibers are locally available and abundant, and their deployment in the development of construction materials consumes lower energy and costs compared to petrochemical-based materials.

**Figure 8:**
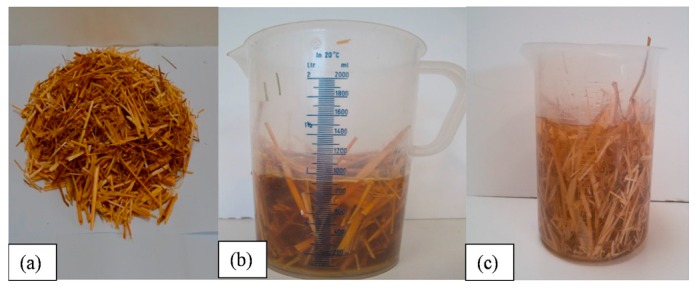
Treatment of straw fibers: (**a**) crushed straw, (**b**) immersion in linseed oil, (**c**) boiled water immersion [46].

Belayachi et al. [46] also investigated the use of wheat and barley straw fibers in the manufacture of light-weight composites for building insulation. However, unlike Rojas et al. [44], a comparison was made between treated and untreated fibers to investigate the impact of treatment on the composites’ fire behavior in terms of flammability and thermal degradation. The fibers were mixed with lime or gypsum plaster in the investigation. Additionally, they were also treated using boiled water and linseed oil in order to decrease water absorption while enhancing the compatibility and adhesion of the binder. Figure 8 below displays the various treatment alternatives adopted.

**Figure 9:**
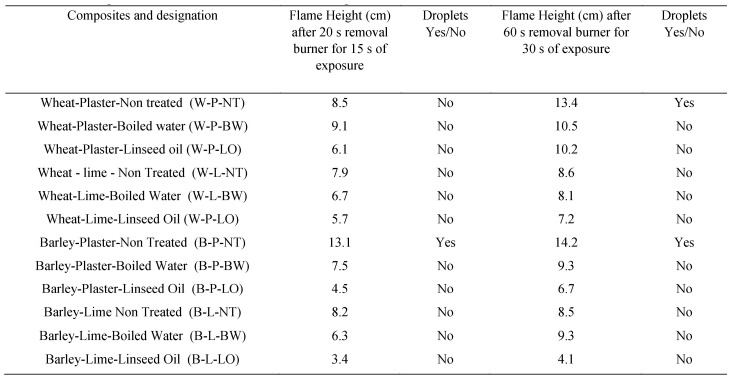
Flame spread after burner exposure for 15 and 30 s [46].

After soaking for different periods (10 s, 5, 10, 15, and 60 min), flammability tests were undertaken for the impregnated composites with analysis being completed using infrared cameras in order to evaluate temperature during and after the exposure of the flames. Figure 9 below further summarizes the results of exposing the different treated and untreated composites to flames.

The findings obtained showed that the linseed oil served as a flame retardant, and in effect, the retarded treatment spread of the flame and further prevented the degradation of the composite [46]. Similarly, further analysis showed that barley-based composites demonstrated delicate fire behavior than wheat composite. The research implies that additional treatment to natural fibers enhances their thermal properties such as flammability and thermal degradation, and therefore, real-world applications should leverage on such insights in insulating buildings.

**Figure 10:**
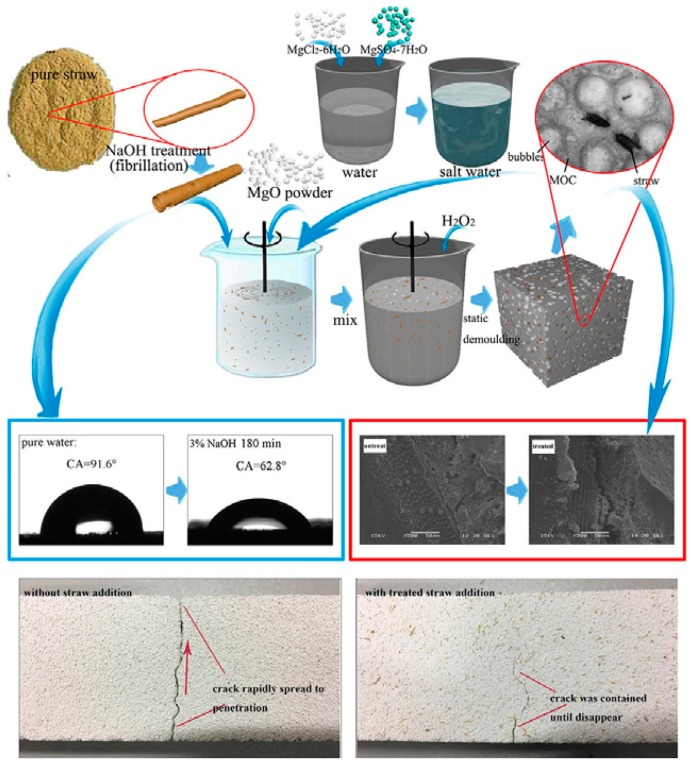
Manufacturing process of the straw/magnesia composite [47].

In separate research, Wang et al. [47] also investigated the thermal and mechanical properties of composites developed from rice straws, magnesium cement adhesive, and a foaming agent. Similar to Belayachi, Hoxha, and Ismail [46], the composites were also treated. However, alkali (NaOH) rather than linseed oil was used. An assessment of the straw properties and bonding between matrix and straw was also undertaken. Figure 10 below displays the graphical abstract of the processes followed during the production of the composites.

The findings obtained showed that the mechanical properties of the composite attained maximum value when the straw was mixed with 3% NaOH for 150 min. Furthermore, compared to other composites, the developed composite was lighter, non-flammable, and provided heat insulation. The research is vital, as it confirms the findings by Belayachi et al. [46] that the treatment of composites manufactured using agro-wastes such as rice, wheat, or barley straws enhances their thermal and mechanical features.

Other researchers expanded the research by investigating the impact of adding other agro-wastes to the cement/straw composites. For instance, Bakatovich and Gaspar [48] investigated the use of Sphagnum moss as a fiber in thermal insulation panels. In the study, several compositions were developed for thermal insulation boards based on rye straw, reed, and moss, while using liquid glass binder. The composites were further assessed for thermal conductivity and physical properties such as compressive strength and bending. The findings obtained showed that optimal results were obtained for insulation panels that combined moss and straw fibers. Good thermal conductivity of 0.044 to 0.046 W/mK and a density of 156–190 kg/m^3^ were recorded.

Similarly, a compression strength between 0.20 and 0.21 MPa was also obtained without any shrinking during the drying process. Similar sentiments were also echoed by Sair et al. [49], who developed eco-friendly composites by mixing a gypsum matrix with two natural fibers: cardboard and cork waste. The findings obtained showed that mixing the two composites with the gypsum building material enhanced the insulation capacity. Likewise, the paper fiber enhanced the compression property of the composite [49]. In a further study, Wang et al. [50] investigated the thermal conductivity and mechanical properties of konjac glucomannan (KGM)/starch-based aerogel enhanced by wheat straw. The wheat straw and starch were added to enhance the physical properties of the aerogel, such as the distribution of pore sizes and mechanical strength. The findings obtained showed that adding starch and wheat straw enhanced the mechanical strength of the composites, whereas wheat straw decreased the pore sizes of the aerogel. Additionally, the optimized aerogel composite was observed to demonstrate low thermal conductivity at 0.046 Wm-1K-1, excellent thermal stability, a compression modulus of 67.5 kPa, and an elasticity of 0.27.

The results reveal that building insulation materials can be enhanced further by incorporating additional agro-waste such as moss fibers, starch, or cardboard. Directly, this suggests that practitioners in the building industry are presented with several alternatives to enhance the sustainability of thermal insulation building materials—on the one hand, treatment using alkalis such as NaOH or linseed oil enhances their thermal features, while on the other, adding other agro-waste materials to the composites also achieves similar qualities.

### 4.4. Reinforcement Materials for Buildings

Research reveals that agro-wastes are also being deployed in the manufacture of reinforcement materials for buildings. However, before examining their use, it is crucial to highlight findings from the previous section, which revealed that building materials developed from agro wastes were light weight. For instance, Taurino et al. [29] revealed that light-weight bricks could be developed by integrating clay and agro-wastes from wine less and grape seeds.

Kimeng et al. [39] also observed that concrete panels cast using groundnut shells as a replacement of aggregate could not be utilized in heavy structural applications due to their lower compressive strength. Belayachi et al. [46] also reported that building insulation composites developed using wheat and barley straw fibers were also light weight in nature. Therefore, in examining the applications of agro-waste-based reinforcement materials, the focus shifts to research that demonstrates how the various types of agro-waste were utilized in reinforcement applications.

**Figure 11:**
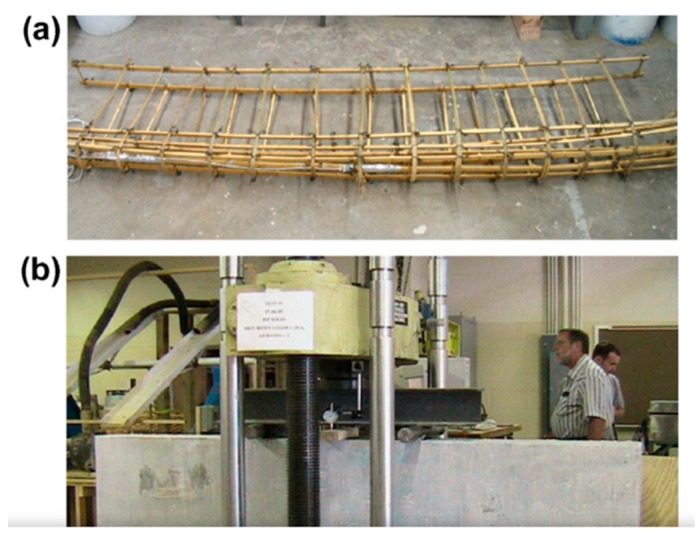
Concrete beams reinforced with bamboo rebars: (**a**) finished reinforcement, (**b**) the setup [51].

To begin with, Pacheco-Torgal and Jalali [51] revealed that using vegetable fibers as reinforcement in cement-based materials enhanced the durability performance and properties of the cementitious materials. Figure 11 below displays concrete beams reinforced with bamboo rebars, which represent vegetable fiber.

**Figure 12:**
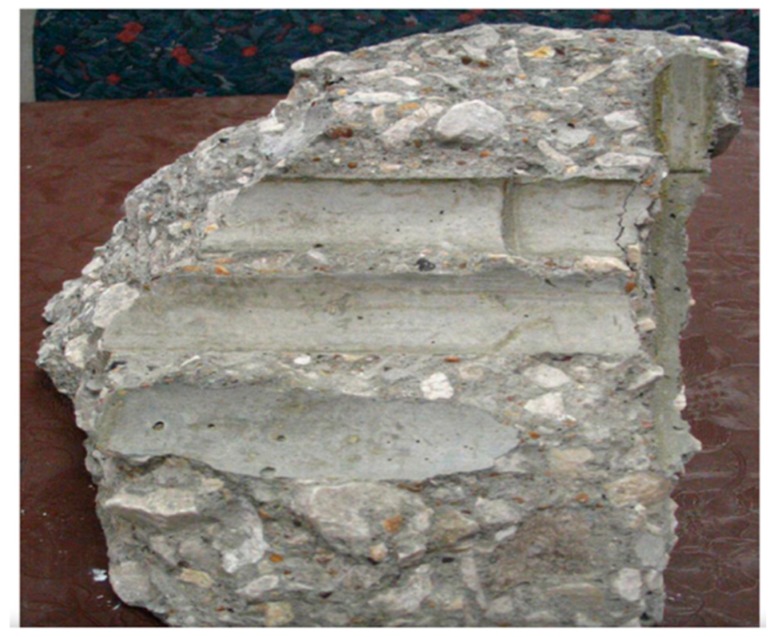
Imprint of bamboo reinforcement in cementitious material [51].

The research also revealed that a wide range of vegetable fibers could be adopted in reinforcing cement-based materials such as sisal, coconut, bamboo, and hemp, among others. Figure 12 illustrates an imprint of bamboo reinforcement in the cementitious material.

In separate research, Sharda et al. [52] also revealed that fiber was an effective alternative to steel bars in reinforcement applications in concrete. The researchers postulated that using fibers in concrete materials improved their durability based on the results of tests, such as freeze–thaw resistance, carbonation depth, and permeability. However, Sharda et al. [52] highlighted that there was a need to exercise caution and ensure competency in the execution of the fiber reinforcement. Guna et al. [3] investigated the use of untreated rice husk (RH) and groundnut shell (GNS) in the preparation of hybrid polypropylene (PP) biocomposites for green building materials. Tests were after that conducted to assess the impact of the percentage of reinforcement on aspects such as sound absorption, flame resistance, thermal insulation, aqueous stability, and mechanical properties. The findings obtained showed that the reinforced composites possessed good flexural and tensile strength with the highest values being recorded as 37.6 MPa and 15.6 MPa respectively for the 20/60/20 GNS/RH/PP ratio.

**Figure 13:**
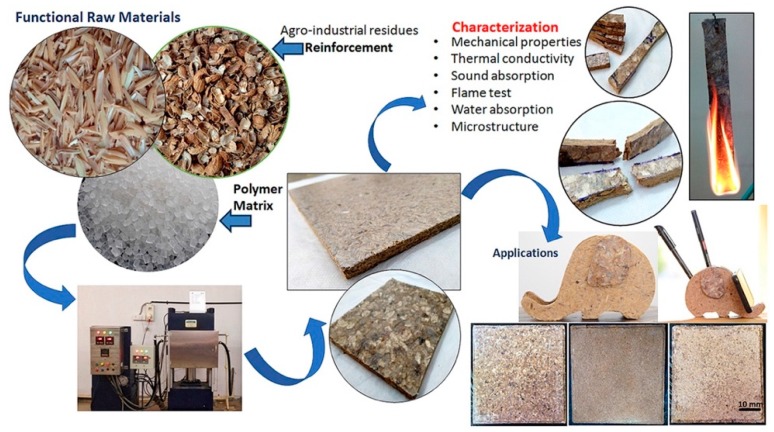
Reinforcement of hybrid polypropylene using rice husk and groundnut shells [3].

Additionally, thermal conductivity was varied from 0.156 to 0.270 W/mK, and the maximum sound absorption coefficient was recorded as 0.48. As such, the properties of the composites were comparable to other biocomposites. Figure 13 illustrates the graphical abstract of the manufacture of the composites.

In a different study, Hassan et al. [53] investigated the mechanical and morphological properties of carbonized maize stalk used to reinforce polyester composites in the manufacture of eco-friendly composites. The carbonized maize stalk ash particles (MSAps) were added in four separate ratios—5%, 10%, 15%, and 20%—and the composite samples produced were analyzed in order to assess the impact of the different ratios. The findings obtained showed that an increase in the maize stalk ash particles resulted in a commensurate increase in the tensile strength, compressive strength, and tensile modulus of the composites. However, the impact strength decreased gradually. The results implied that MSAps could be used to enhance the polymer matrix composites used in building and automobile applications.

Natali et al. [54] further investigated the use of fiber to reinforce geopolymer composites due to their poor tensile and bending strengths, which resulted in their brittle and ceramic nature. The findings obtained also showed that both organic and inorganic fibers enhanced the flexural strength of the geopolymers. The geopolymers were also observed to increase their toughness [54]. In a separate study, Santos et al. [55] investigated the reinforcement of a polymeric matrix by utilizing untreated natural fibers based on PLA and pine resin added at three different ratios: 5%, 7.5%, and 10%. The morphological and thermal characteristics of the fiber were further evaluated. The findings showed that there was an increase in the flexural strength of composites that had natural fibers added as compared to those that excluded them.

Yang et al. [56] also comparatively investigated the possibility of using waste tire composites reinforced with rice straw as composite construction materials. In the study, two types of panel insulation boards were manufactured: wood-particle based and composite boards made by mixing agricultural lignocellulosic fiber (rice straw) waste tire particles at parameters of specific gravity of 0.8 and rice straw content in ratios of 10/90, 20/80, and 30/70 by weight of the rice straw in the insulation boards. Polyurethane adhesive for rubber was used as the composite binder. The findings obtained showed that the composite boards reinforced by the rice straw wastes demonstrated better water absorption, waterproof qualities, and thickness swelling than the wood particleboard. The composite boards were also more superior to the wood-based and had good acoustical insulation, anti-rot, anti-caustic, and electrical insulation features. Similarly, the research showed that the composite boards could be used as an adequate replacement for insulation boards and other flexural construction materials due to their excellent qualities in preventing impact damage, their inexpensiveness, and ease of modification.

A side-by-side assessment of the findings highlights two critical findings. First, agro-waste materials could be used in reinforcement applications in their natural form, for instance, bamboo in cementitious applications. Secondly, the agro-waste materials could be treated or used in chemical admixtures in reinforcing biocomposites, thereby suggesting that treating the agro-waste was necessary before utilizing them in reinforcement applications. Nevertheless, a confluence observed with the different studies was that the building materials developed demonstrated excellent properties, which were comparable with other conventional materials.

**Figure 14:**
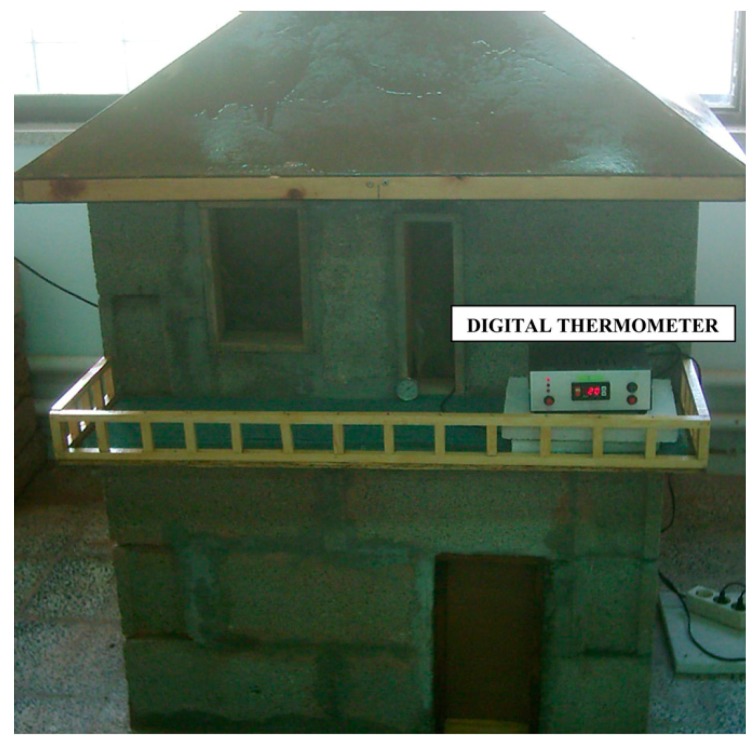
Model house built with fiber-reinforced mud bricks [57].

In other instances, some researchers investigated the use of natural fiber in reinforcing other construction materials such as bricks. For instance, Binici et al. [57] investigated the impact of reinforcing mud bricks using two types of fiber: plastic fibers, such as polystyrene, and agro waste, such as straw. The findings showed that bricks reinforced with the natural fibers attained the heat conductivity and compressive strength requirements established by the ASTM and Turkish Standards. Additionally, Basaltic pumice was used as an ingredient to lower the thermal conductivity coefficient of fiber-reinforced bricks. However, upon the comparison of the two reinforced bricks, mud bricks reinforced with plastics demonstrated higher compressive strength than both those without fibers and those that used straw. Nonetheless, the study concluded that mud-brick houses reinforced by fiber were superior to concrete houses in terms of maintaining indoor temperatures during winter and summer seasons. Figure 14 below illustrates a model house built with fiber-reinforced mud bricks.

Saxena et al. [58] further investigated the potential of using reinforced polymer composites as a substitute for conventional wood materials. The findings showed that composites developed using natural fiber or industrial wastes, such as red mud and fly ash, attained superior mechanical properties and were also resistant to fire, abrasive wear, chemical attack, and water absorption as compared to conventional products such as wood, particleboards, and medium density fiber (MDF). The researchers further argued that plant fiber and industrial waste polymer composite materials were able to serve as a potential substitute for conventional wood due to their savings on cost and energy and their technological viability. An additional implication of the findings is that engineers are now able to explore the use of readily available agro-waste in the development of construction materials, as this was excluded in previous decades [58].

### 4.5. Particleboards

**Figure 15:**
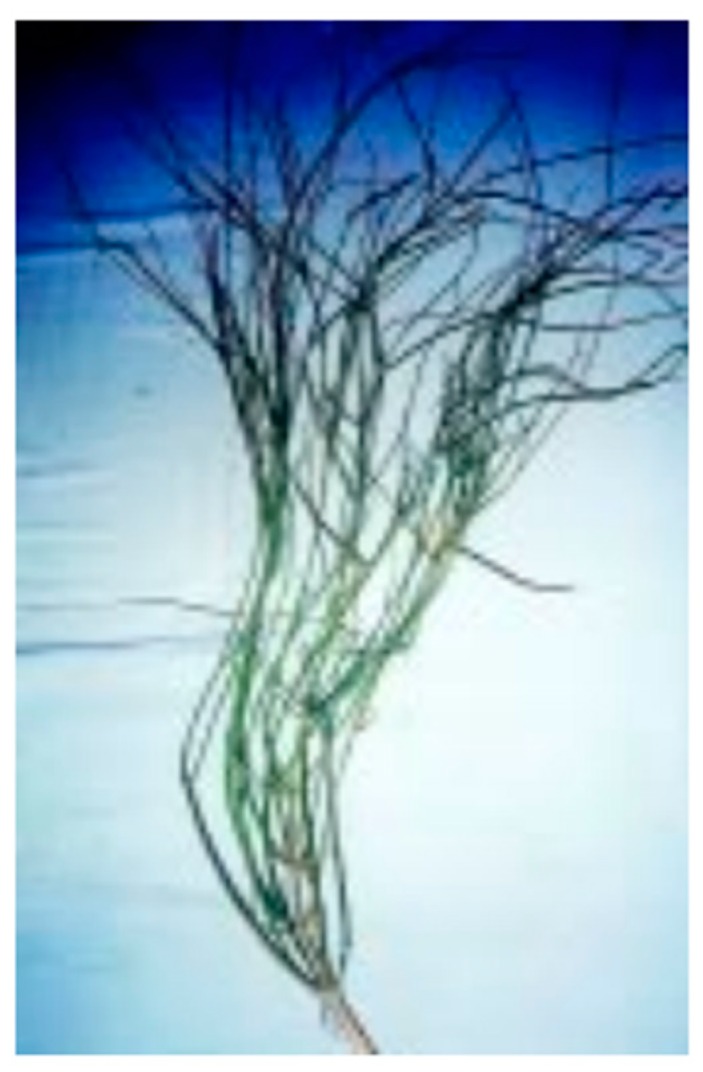
Khimp plant shrub [59].

Particleboards or chipboards are engineering wood products developed from the compression of wood chips, sawdust, or wood shavings bound with synthetic resin. Research shows that agro-waste is also used in the manufacture of particleboards as a substitute for conventional wood by-products, such as shavings and sawdust, thereby enhancing the overall product. For instance, Bhaduri and Mojumder [59] investigated the use of the Khimp plant, which is a shrub widely grown in the Rajasthan desert, in the manufacture of medium-density particleboards (60 cm × 60 cm × 1.3 cm) using urea–formaldehyde (UF) and phenol–formaldehyde (PF) resins as binders separately. Figure 15 shows the Khimp plant.

The concentrations of the UF and PF varied from 10% to 20% to assess the impact of adhesive on the physical and mechanical properties of the particleboard. Standard methods were after that employed to evaluate the physical properties of the boards, their density, moisture content, swelling values, water absorption, and mechanical features, such as tensile and impact strength, modulus of rupture, and flex modulus. The findings obtained showed that the physical and mechanical properties of boards developed using UF and PF resins adhered to the established BIS specifications for medium-density general purpose particleboards. However, a key finding noted was that the boards developed using the PF resin demonstrated better physical and mechanical features than UF boards. The economic implication of the study was that wood could be effectively replaced as a raw material for particleboards in the desert region without any adverse impact on the environment. However, the study is unique as it focuses on harnessing desert plants such as the Khimp shrub in the manufacture of building materials.

In other studies, researchers have investigated the potential of replacing wood by-products with agro-waste materials in the manufacture of medium-density particleboards. For instance, Das et al. [60] revealed that particleboards for use in different applications could be manufactured from jute stick agro waste. In a separate study, Das and Chanda [61] further investigated the use of sugar beet leaf residues in the manufacture of particleboards after protein extraction. In the study, several binder systems were utilized: urea–formaldehyde (UF) resin, phenol–formaldehyde (PF) resin, and in situ condensation of formaldehyde and urea without applying any resin. Then, the different boards were tested for physical properties such as moisture content, water absorption, density, and swelling or thickness values.

**Figure 16:**
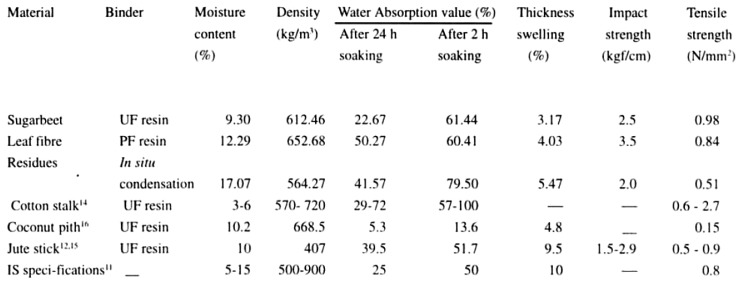
Physicomechanical features of different particleboards [61].

Similarly, mechanical features such as the impact and tensile strength were also calculated based on BIS standards. The findings obtained showed that the properties of boards prepared using the leaf fiber of sugar beet residues demonstrated similar structures to other conventional lignocellulosic materials used in the manufacture of particleboards such as cotton stalk, jute stick, and bagasse [61]. Likewise, a significant difference in the results was also observed due to the use of different binder resins—in particular, boards that were prepared by using an in situ condensation of formaldehyde and urea without resin were inferior to those developed using UF and PF resins. Nevertheless, the study showed that particleboards developed using sugar beet leaf residues adhered to BIS standards for general purpose applications such as cladding, paneling, and indoor partitioning. Figure 16 below illustrates a comparison of the physicomechanical properties of particleboards prepared using different binder systems.

From the results, it was observed that the particleboards from sugar beet leaf fiber manufactured in PF resin demonstrated excellent results in terms of density, thickness, impact strength, and tensile strength.

Rosario et al. [62] also investigated the use of cotton stalks in the manufacture of particleboards using urea–formaldehyde as a binder. In the study, the researcher evaluated the impact of three different resin contents—8%, 10%, and 12%—as well as three different board densities—400 kg/cm^3^, 500 kg/cm^3^, and 600 kg/cm^3^ on the quality of single-layer and three-layered particleboards produced using cotton stalks. The findings obtained showed that only the density of the boards had a significant influence on the quality of particleboards as the heaviest particleboard (600 kg/cm^3^) adhered to PHILSA mechanical standards at any percentage of resin. However, in regard to the dimension stability, only the three-layered particleboards with a density of 600 kg/cm^3^ with resin content at 10% and 12% adhered to established PHILSA standards. Further deductions showed that the optimum three-layered particleboards were developed at 10% resin and a density of 600 kg/cm^3^, whereas single-layered boards were manufactured at 8% resin and a density of 600 kg/cm^3^.

In a different study, Khanjanzadeh et al. [63] investigated the physical and mechanical properties of wood-based three-layered particleboards containing different quantities of cotton stalks and underutilized paulownia wood particles. The two were mixed in ratios of 30%, 50%, and 70% using urea–formaldehyde resin. The findings obtained showed that the mechanical properties of the boards were significantly enhanced after the addition of cotton stalk and paulownia wood. However, on the contrary, higher cotton stalk and paulownia wood particle contents decreased the water resistance capability of the composites. Excellent mechanical properties of the composites were obtained at ratios of 50% and 70%, respectively. Several similarities and differences can be deciphered from the comparison of Rosario et al. [62] and Khanjanzadeh et al. [63], who both used cotton stalks in the manufacture of particleboards. A confluence is that both researchers used urea–formaldehyde as the resin binder and attained relatively similar results in that the boards adhered to established standards. However, a difference that emerges is that Khanjanzadeh et al. [63] demonstrated that the mechanical and physical properties of the particleboards could be enhanced further by adding paulownia wood particles. As such, this suggests that mixing more than one type of agro-waste could enhance the quality of the particleboards.

In order to confirm this assertion, it is imperative to examine other studies where researchers combined more than one type of agro-waste in manufacturing particleboards. A study by Fiorelli et al. [64] comparatively evaluated the thermal–physical–mechanical properties and microstructural properties of multi-layer particleboards that had been manufactured using sugarcane bagasse in their outer layers and an admixture of sugarcane bagasse and Amazonian vegetable fibers such as jute and curaua. X-ray diffraction was used to measure the density of the boards in order to ensure it was maintained at 550 kg/m^3^. The Brazilian standards, ABNT NBR 14,810:2013, were also used to assess the mechanical and physical properties of the boards. Furthermore, the ISO 8301:1999 was applied in order to determine thermal conductivity. The findings obtained showed that the mechanical properties of the particleboards using the admixture containing vegetable fibers were superior to those that only used sugarcane bagasse. Additionally, the thermal conductivity of the different boards was observed to be similar, thereby suggesting that the integration of vegetable fibers did not affect the particular property.

Akinyemi et al. [65] also echoed similar sentiments in a study examining the durability and strength features of particleboards made from wood wastes and expanded polystyrene foam (EPS). In the study, two different wood particle sizes and two different dosages of EPS resins were used as binders in the boards. The modulus of rupture (MOR) and modulus of elasticity (MOE) was after that evaluated. The findings obtained showed that a decrease in the water absorption rates of the composite followed after an increase in EPS content. However, the optimal performance of the boards was recorded before prolonged immersion in water. The study revealed that wood and EPS wastes could be utilized in the production of composite wood panels that could withstand moist environmental conditions.

### 4.6. Bio-Based Plastics

The last construction material discussed is the manufacture of bio-based plastics using agro-wastes. To begin with, Ashori and Nourbakhsh [66] investigated the potential of using agro-waste residues such as sunflower stalk, bagasse fibers, and corn stalk as reinforcement in the manufacture of thermoplastics as an alternative to wood fibers. In the study, two grades of coupling agents—Eastman G-3003 and G-3216—were utilized, and their impact on the mechanical properties of the thermoplastics was assessed. A one-level fiber loading by weight (30%) was used, while three levels of coupling agent contents (0%, 1.5%, and 2.5%) were used as well. The findings obtained showed that where the samples were treated, using either of the two coupling agents, an enhancement in the tensile, flexural, and impact properties was observed as compared to the untreated samples. Likewise, the coupling agent was observed to affect interfacial bonding positively. However, composites treated with the G-3216 agent outperformed those that had been treated using G-1303 due to the high melt viscosity of the G-3003. Samples that utilized bagasse fiber were also observed to demonstrate superior qualities due to their inherent chemical properties.

Leceta et al. [67] also investigated the environmental impact of bio-based films developed from agro-industrial wastes and marine residues. The agro-wastes that were used included soy protein, a by-product of the soy industry, chitosan from the exoskeleton of crustaceans, and agar from seaweeds. The findings obtained showed that unlike polymers manufactured from oil refinery processes, bio-based films made from agro-wastes and marine residues exerted the least negative impact on the environment due to their biodegradable characteristics. Lambert and Wagner [68] also evaluated the manufacture of bio-based plastics from a similar angle in their review paper whereby specific focus was directed toward their degradation in the environment. However, the researchers revealed that the integration of food wastes and microalgae had an impact on the polymer structure and in effect influenced the performance of the composites in addition to impacting biodegradability [68].

Ashok et al. [69] have further argued that bioplastics are superior to conventional plastics derived from fossil fuels in terms of energy efficiency, carbon emission, and the consumption of petroleum. However, the researchers reveal that the bioplastics are inferior in terms of their applicability. To that end, they proposed to integrate starch in the manufacture of bioplastics in order to further enhance properties such as the hardness, impact strength, and biodegradability. In a separate study, Gonçalves de Moura et al. [70] employed green chemistry methodology to extract natural polymers such as cellulose from vegetable wastes. As a result, the researchers were able to prepare new functional polymers for packaging based on the extracted cellulose, which had demonstrated solid thermomechanical properties and biodegradability. The implication of the research was its revelation of the potential of agro-waste in the production of new cellulose-based plastics for applications in food packaging [70]. Analysis of the three studies [69,70,71] reveals that the utilization of agro-waste in the manufacture of bio-based plastics not only enhances their inherent mechanical properties, such as impact strength but also improves their biodegradability, thereby alleviating the adverse environmental pollution challenge.

Chiellini et al. [72] also demonstrated the preparation of environmentally degradable blends and composites under different conditions. In the study, polyvinyl alcohol (PVA) was used as the synthetic polymer of choice due to its ease of processing from water solutions or suspensions as well as being melted by injection molding and blow extrusion. Additionally, starch and gelatin were utilized as the polymeric materials and natural fillers such as sugarcane bagasse (SCB), apple peels (AP), corn fibers (CF), wheat flour (WF), wheat straw (WS), and orange peels (OP) used. Similar to previous studies [69,70], the researchers also observed that the integration of agro-waste in the production of bio-based polymeric materials and fillers enhanced their morphological and mechanical properties and also resulted in biodegradable end products that could not affect the environment adversely.

## 5. Advantages and Disadvantages of Agro-Waste Construction Materials

Based on the comprehensive evaluation of the different construction materials manufactured using agro-waste, as described in the previous sections, several advantages and disadvantages are identified. In this section, a summary of the benefits and shortcomings associated with the utilization of agro-wastes in developing building materials is performed.

### 5.1. Advantages of Using Agro-Waste in Manufacture of Construction Materials

The advantages of utilizing agro-waste in the manufacture of construction materials are classified into two main groups. First are the advantages that arise from reusing waste agro-waste materials that would otherwise be disposed to the environment; second are the advantages that arise from the use of the agro-waste materials in the actual development of building materials.

With the first category, a significant advantage stems from the fact that reusing the agro-waste in manufacturing processes helps tackle the pollution challenge that arises from conventional disposal approaches such as dumping in landfills, incineration, and composting [8]. Research showed that different agricultural processes resulted in the generation of significant waste levels that introduced challenges in the disposal and proper management. For instance, in India, over 600 metric tonnes (MT) of waste was reported from agricultural-based products alone [2]. Therefore, reusing the waste materials implies that fewer resources are used in managing agro-waste programs, as fewer quantities require to being disposed of. A second advantage relates directly to the environment, whereby as agro-waste materials are reused as alternatives to conventional building materials such as cement or sand aggregates, less non-renewable resources are exploited to facilitate production processes. As such, this leads to environmental conservation, as less energy is also consumed in producing the building materials.

With the second category, the review paper also identified several advantages. To begin with, in the construction of brick or masonry components, the use of agro-waste led to quality light-weight bricks that adhered to established construction standards. Kazmi et al. [27] showed that utilizing rice husk ash (RHA) and sugarcane bagasse ash (SBA) in producing clay bricks, by incorporating 5% of the RHA and SBA by clay weight led to bricks whose modulus of rupture and compressive strength adhered to Pakistan building codes and ATSM standard guidelines. Taurino et al. [29] also demonstrated the manufacture of light-weight bricks by integrating wine wastes (WWs) such as wine less, grape seeds, and stalks with clay. The different studies showed that the agro-waste had potential in developing building materials that adhered to required construction standards.

Similarly, with the green concrete, diverse researchers showed that agro-waste facilitated the development of construction materials that were cheaper and highly effective. An analytical study by Akram et al. [38] demonstrated the use of sugarcane bagasse ash (SCBA) as a viscosity-modifying agent in self-compacting concrete. Directly, this has a significant economic implication, as it eliminates the cost of hiring vibrators to undertake compaction processes.

With the insulation materials, the use of agro-wastes such as hemp revealed that the biocomposite had excellent insulation properties and mechanical resistance [42]. In effect, this implied that the inhabitants of houses insulated using building materials developed using agro-waste derived comfort at a much affordable cost. Therefore, insulation materials lead to both social and economic sustainability. On the other hand, using agro-waste for the development of reinforcement materials showed that the durability and performance were improved significantly. For instance, Pacheco-Torgal and Jalali [51] revealed that using vegetable fibers as reinforcement in cement-based materials enhanced the durability performance and properties of the cementitious materials. In separate studies, research also showed that using carbonized maize stalk ash particles enhanced the tensile strength, compressive strength, and tensile modulus of the composites. Therefore, the advantages of the enhancement of building materials are further associated with the integration of agro-waste variants.

Finally, with the use of agro-waste to develop particleboards and bio-based plastics, diverse advantages of economic and environmental sustainability were also identified. For instance, with the particleboards, using alternatives such as the Khimp plant shrub led to suitable quality materials that adhered to BIS standards [59]. Directly, this suggests that a shift is expected away from wood by-products, thereby reducing the adverse environmental impact and promoting the economic benefit of using such materials. With the bio-based plastics, research showed that they were superior to conventional plastics derived from fossil fuels in terms of energy efficiency, carbon emission, and the consumption of petroleum [69]. Furthermore, it also improved environmental sustainability due to its biodegradable nature.

In summary, it can be argued that the different agro-waste-based construction materials generated three main types of advantages: economic, environmental, and social sustainability. As their production required less energy, this implied less costs and resources to produce them. Likewise, the environment would also be conserved from the use of insulation materials that reduced energy demands. Inhabitants of the different buildings would also attain comfort and maximum quality by living in functional quality environments.

### 5.2. Disadvantages of Using Agro-Waste in Manufacture of Construction Materials

Despite the numerous advantages associated with the use of agro-waste in the development of construction materials, several shortcomings were also identified. However, only a few challenges were identified, with the primary factor being the light-weight nature of the agro-waste-based construction materials. For instance, with the brick/masonry-making applications, a key disadvantage observed was that the bricks generated from the manufacturing processes were light weight, and as such, they were only suitable for some structural applications. For instance, Kazmi et al. [27] argued that bricks developed by using rice husk ash (RHA) and sugarcane bagasse ash (SBA) and clay could only be used in applications where lower structural loads were required in buildings. A similar finding was also observed with the insulation materials for buildings where Benfratello [42] argued that the admixture of hemp and lime concrete was lighter than conventional concrete, and as such could only be used appropriately where lower loads in buildings were required—for instance, in the green coverings of the top of an existing building. However, despite the light-weight nature of some construction materials such as bricks, other agro-waste applications were nonetheless observed to support heavier applications, for instance, in reinforcement applications. Therefore, the argument that agro-waste-based construction materials are only suited for light-weight applications such as rooftops [42] or other lighter structural elements [27] is not valid. For instance, Shardaet al. [52] demonstrated the use of fiber as an effective alternative to steel bars in reinforcement applications in concrete. The researchers postulated that using fibers in concrete materials improved their durability based on the results of tests such as freeze–thaw resistance, carbonation depth, and permeability. In a separate study, Natali et al. [54] further showed that reinforcing geopolymer composites by adding either organic or inorganic fibers enhanced their flexural strength as well as their toughness. The separate research studies reveal that agro-waste-based construction materials can be adopted in both light weight and more robust applications, and as such, their disadvantage is relative to the applications that they are put into. For instance, where the focus is to develop agro-waste-based bricks, the materials would be disadvantageous as they are only suited in light-weight applications. However, using natural fibers in concrete would be an added advantage, as they improve the durability of the materials.

Another disadvantage further observed was the need for high expertise in their manufacture. For instance, Sharda et al. [53] highlighted the need to exercise caution and ensure competency in the execution of natural fiber reinforcement in cementitious materials. Similarly, this is a relative disadvantage based on the availability of highly skilled labor in developing the materials. Where skilled labor is affordable and readily available, construction projects are developed quickly compared to scenarios where the labor has to be sourced more expensively. A third disadvantage can also be identified in the requirement of multiple pre-processes before the agro-waste could be used in actual construction projects. For instance, as revealed in Figure 10, composites made from rice straws had to be treated using alkali (NaOH) before mixing with an adhesive and foaming agent [47]. Likewise, Belayachi et al. [46] had also shown that in manufacturing composites for building insulation from wheat and barley straw fibers, several pre-processes had to be undertaken such as mixing with lime or gypsum plaster and treating the straws with boiled water or linseed oil in order to decrease water absorption while enhancing the compatibility and adhesion of the binder. While it could be argued that the pre-process treatments were required for experimental purposes, in large-scale applications, such treatments are costly and require time, thereby being disadvantageous.

Additional challenges and shortcomings were also linked to the production of the sustainable construction materials. To begin with, the analysis of the brick production processes showed that conventional approaches were easier to implement as bricks were developed by first mixing earth-based materials and afterwards, the bricks were molded, dried, and fired in kilns. On the other hand, developing the sustainable bricks was observed to be more challenging, particularly since the researchers did not have guidelines on how to integrate agro-waste with the conventional materials. For instance, Kizinievič et al. [28], in developing sustainable clay bricks by adding oat and barley husk middlings, had to prepare three separate concentrations—5%, 10% and 20%—and compare the results obtained. Likewise, De Silva and Perera [30] in preparing clay bricks by adding rice husk ash (RHA) had to mix the agro-wastes in different concentrations: 0%, 2%, 4%, 6%, 8%, and 10%. Similar findings were also observed in the preparation of green concrete where Modani and Vyawahare [35] replaced untreated sugarcane bagasse ash by volume of fine concrete aggregate in ratios of 0%, 10%, 20%, 30%, and 40%. Hassan et al. [53] also added carbonized maize stalk ash particles (MSAps) in four separate ratios—5%, 10%, 15%, and 20%—in the reinforcement of polyester composites. The analysis showed that there lacked guidelines to facilitate the development of various sustainable construction materials, thereby leading to mixing in different concentration ratios. Directly, this highlights an existent research gap to investigate and identify optimal ratios that should be adopted in the integration of different types of agro-waste. Indirectly, however, it suggests that experimentation is still needed in real-world applications where the agro-waste-based construction materials are needed. Subsequently, this leads to higher costs and the utilization of more time before determining the optimal ratios that should be used.

A further production challenge regards the pre-processing phases involved with some agro-wastes before they could be integrated with conventional materials. Belayachi et al. [46], in manufacturing light-weight composites for building insulation, mixed wheat and barley straw fibers that were treated using boiled water and linseed oil in order to decrease water absorption while enhancing the compatibility and adhesion of the binder. Wang et al. [47], in developing composites from rice straws, magnesium cement adhesive, and a foaming agent, also treated the agro-wastes using alkali (NaOH). Similarly, in the manufacture of brick and masonry components, the researchers were also observed to burn the different solid wastes, such as groundnut, rice, and barley husks in order to generate ash that could be integrated with conventional materials. Additionally, the research showed that the ash had to be sieved further before being integrated to clay bricks. Refer to Figure 3, which shows the processes used to generate SCBA where the solid waste was burned to generate ash. The direct implication of the pre-processing activities is that they suggest that manufacturing sustainable construction materials requires more resources in the long run in order to cater for the pre-processing required, for instance, treatment with chemicals and burning solid waste to generate required ash. However, in other instances, researchers used the raw agro-waste without any additional processing. For instance, Pacheco-Torgal and Jalali [51] used vegetable fibers as reinforcement in cement-based materials as shown in Figure 12. This also suggests that additional processing is not a mandatory requirement for all types of agro-based construction materials.

## 6. Discussion

In this review paper, the researcher focused on examining the various applications of agro-waste in the development of sustainable construction materials. Six different materials were reviewed in this regard: brick/masonry elements, green concrete, insulation materials for buildings, reinforcement materials for buildings, particleboards, and bio-based plastics. Based on the evaluation of the different articles, several noteworthy findings are outlined. First, with the brick/masonry materials, the bricks generated by mixing agro-waste with clay were light weight in nature due to the porosity and low compressive strength of the agro-waste. However, the light-weight nature could not be eliminated by increasing the ratio of agro-waste, since the optimal performance of the bricks was only identified at low ratio percentages. For instance, De Silva and Perera [30] showed that optimal bricks were only attained at 4% RHA ratios, while Kazmi et al. [27] identified a 5% RHA ratio as the optimal percentage for the bricks. The low ratio agro-waste aspect was also identified with the green concrete production: for instance, Rao and Prabath [36] and Modani and Vyawahare [35] obtained high-quality bricks at 10% sugarcane bagasse ash (SCBA) ratios.

Secondly, it was further observed that mixing more than one type of agro-waste material in the manufacture of construction materials led to improved performance and qualities of the deliverables. For instance, Khanjanzadeh et al. [63] demonstrated that the mechanical and physical properties of the particleboards could be enhanced further by adding paulownia wood particles. Likewise, Fiorelli et al. [64] showed that the mechanical properties of the particleboards using an admixture containing vegetable fibers were superior to those that only used sugarcane bagasse. With the green concrete production, it was also observed by Rodier et al. [37] that cement pastes made from ternary mixtures of bamboo leaves ash (BLA) and sugarcane bagasse ash (SCBA) had higher pozzolanic activity than the binary mixture of SCBA only. Fiorelli et al. [64] further showed that manufacturing multi-layer particleboards using an admixture of sugarcane bagasse and Amazonian vegetable fibers such as jute and curaua produced boards with better mechanical properties than those produced using only sugarcane bagasse in their outer layers.

Similarly, Binici et al. [57] had shown that reinforcing mud bricks using natural fibers improved their heat conductivity and compressive strength, attaining requirements established by the ASTM and Turkish standards. However, in contrast, the researchers showed that mud bricks reinforced using plastics led to higher compressive strength than in cases where bricks were not reinforced with any fibers or where natural fibers such as straw were used. The findings highlight a research gap in investigating the underlying factors, leading to the improvement of the performance and qualities of agro-waste-based construction materials following the incorporation of more than one agro-waste material.

A third important finding regards the adherence of agro-waste-based construction materials to sustainability requirements. First of all, it is essential to highlight that most of the researchers observed that the construction materials developed using agro-waste materials complied with existent building standards. For instance, with the bricks, Deraman et al. [31] showed that clay bricks developed using 5% concentration of empty fruit bunch (EFB) and coconut fiber (CB) as an additive poring agent generated bricks that adhered to BS392:1985 in terms of water absorption and compressive strength as well as the ASTM C517 thermal conductivity standard. Likewise, Mandal et al. [31] revealed that insulation bricks developed by blending sawdust (7.5%) and 40% red mud at 1100 °C were able to meet the criterion of Type A insulation bricks specified by IS:2042. Binici et al. [57] further showed that reinforcing mud bricks using natural fibers led to the attainment of the heat conductivity and compressive strength requirements established by the ASTM and Turkish standards. The findings directly suggest that agro-waste-based materials are economically sustainable, since they use low-cost production materials (agro-waste) and adhere to the requirements of high quality. A different argument advanced is that the construction materials meet environmental sustainability requirements, as they facilitate the conservation of natural resources by providing an alternative to non-renewable cement and sand aggregates, among others. Additionally, it can be further argued that the construction materials meet social sustainability demands as they promote employment opportunities for local communities that reuse the agro-waste materials in developing the building materials.

Several research gaps also emerge from this study. First, it emerged that low concentrations of agro-waste materials were required as additives in the manufacture of different construction materials. For instance, in the manufacture of bricks and green concrete, a concentration of agro-waste ranging from 4% to 10% was observed to generate high-quality materials. Similar findings were also observed with the manufacture of particleboards. These findings suggest an open area for future research work in order to determine why lower concentrations produce optimal results, whereas increasing agro-waste concentration does not improve quality or performance. Second, as previously highlighted, there is a need for further research to determine why integrating more than one type of agro-waste material enhanced the qualities of the construction materials. Third, despite the high evidence showing the adherence of agro-waste-based materials to existent building quality standards, there is a need to undertake follow-up research to identify the different issues that arise in the post-implementation phases. Empirical investigations are required to identify the performance of the materials in the long run and how they interact with natural elements such as weather, climate, and human use.

## 7. Conclusions

In conclusion, the review paper has examined the utilization of agro-waste materials in the production of sustainable construction materials. A comprehensive review of different research studies showed that the integration of natural organic waste from agricultural processes such as sugarcane bagasse, rice husks, and groundnut shells among others in the manufacture of different construction materials improved their overall physicomechanical and thermal qualities and also enhanced their sustainability properties by reducing costs and boosting environmental conservation. Furthermore, the findings also showed that higher durability advantages also emerged from the use of agro-waste-based construction materials.
